# Protein for Life: Review of Optimal Protein Intake, Sustainable Dietary Sources and the Effect on Appetite in Ageing Adults

**DOI:** 10.3390/nu10030360

**Published:** 2018-03-16

**Authors:** Marta Lonnie, Emma Hooker, Jeffrey M. Brunstrom, Bernard M. Corfe, Mark A. Green, Anthony W. Watson, Elizabeth A. Williams, Emma J. Stevenson, Simon Penson, Alexandra M. Johnstone

**Affiliations:** 1 Rowett Institute, School of Medicine, Medical Sciences and Nutrition, University of Aberdeen, Ashgrove Road West, Aberdeen AB25 2ZD, UK; marta.lonnie@abdn.ac.uk (M.L.); emma.hooker@abdn.ac.uk (E.H.); 2 National Institute for Health Research, Bristol Biomedical Research Centre, University Hospitals Bristol NHS Foundation Trust and University of Bristol, 12a Priory Road, Bristol BS8 1TU, UK; jeff.brunstrom@bristol.ac.uk; 3 Department of Oncology & Metabolism, The Medical School, The University of Sheffield, Beech Hill Road, Sheffield S10 2RX, UK; b.m.corfe@sheffield.ac.uk (B.M.C.); e.a.williams@sheffield.ac.uk (E.A.W.); 4 Insigneo Institute for in silico medicine, The Pam Liversidge Building, Mappin Street, Sheffield S1 3JD, UK; 5 Department of Geography and Planning, School of Environmental Sciences, University of Liverpool, Liverpool L69 7ZT, UK; mark.green@liverpool.ac.uk; 6 Human Nutrition Research Centre, Institute of Cellular Medicine, Medical School, Newcastle University, William Leech Building, Newcastle upon Tyne NE2 4HH, UK; anthony.watson@newcastle.ac.uk (A.W.W.); emma.stevenson@newcastle.ac.uk (E.J.S.); 7 Campden BRI, Station Rd, Chipping Campden, Gloucestershire GL55 6LD, UK; simon.penson@campdenbri.co.uk

**Keywords:** ageing, appetite, older adults, plant proteins, protein, sarcopenia, sustainability

## Abstract

With an ageing population, dietary approaches to promote health and independence later in life are needed. In part, this can be achieved by maintaining muscle mass and strength as people age. New evidence suggests that current dietary recommendations for protein intake may be insufficient to achieve this goal and that individuals might benefit by increasing their intake and frequency of consumption of high-quality protein. However, the environmental effects of increasing animal-protein production are a concern, and alternative, more sustainable protein sources should be considered. Protein is known to be more satiating than other macronutrients, and it is unclear whether diets high in plant proteins affect the appetite of older adults as they should be recommended for individuals at risk of malnutrition. The review considers the protein needs of an ageing population (>40 years old), sustainable protein sources, appetite-related implications of diets high in plant proteins, and related areas for future research.

## 1. Introduction

In the UK, it is projected that by 2035 the majority of the population will be aged 40 or older [1]. The considerable size of this cohort has seen increasing interest from policy officials in utilizing dietary guidance to maintain or improve their health and wellbeing to promote healthy ageing. Adequate intake of protein is one of the key nutritional factors to maintain independence, predominantly by preventing loss of muscle mass and strength (sarcopenia), frailty and associated comorbidities in later life [2,3,4]. At present, the food sector is failing to identify and directly address the needs of this ageing population, with affordable, palatable and practical food solutions.

It is debatable at what exact point in life muscles start to age. A gradual decline in muscle mass is observed from the third decade of life [5], with a 30–50% decrease reported between the ages of 40 and 80 [6]. Muscle strength is correlated with muscle mass and rapidly declines after the age of 50 [7,8]. The beginning of the fourth decade of life might therefore be interpreted as the time when muscle ageing process begins and for this reason it is the optimal time for implementing appropriate dietary changes, to prevent or delay the onset of sarcopenia. Accordingly, throughout this review an ‘ageing adult’ is used to refer to a person aged 40 and older.

Previous work has focused on identifying the optimal protein amount, timing and type of protein for sarcopenia prevention. A number of studies have found that intake exceeding the Recommended Daily Allowance (RDA) may be preferential in preserving muscle mass and functions in ageing adults [3,9,10,11,12]. In addition, the pattern of protein consumption was suggested to be of greater importance than the total daily amount consumed [13], which will be discussed in the next section. The evidence from these studies is however limited to investigating the effects of different types of animal proteins on muscle health [14,15], and the effects of plant proteins (other than soy) have not been adequately studied. 

Plant-based nutrition has received much attention in the past decade [16,17]. The ever-growing demand for foods naturally rich in protein is part of an ecological debate around whether more sustainable sources should be encouraged [18]. The high proportion of animal-protein consumption in developed countries [19] raises both health and environmental concerns. Firstly, dietary patterns characterised by a high intake of animal protein have been associated with increased risk of obesity, diabetes, cardiovascular disease mortality and some cancers [20,21,22,23]. However, it has to be stressed, that dietary patterns describe diet as a whole and it cannot be concluded that all components (e.g., meat, fish, eggs, dairy) of animal-based patterns have an equal, detrimental effect on health. Secondly, animal-protein consumption requires large areas of dedicated land, water, nitrogen, and fossil energy for production and transportation [24,25]. The result is the emission of large amounts of greenhouse gases (GHG) [26]. The health benefits of plant-proteins (as a more sustainable alternative) in sarcopenia prevention have yet to be investigated extensively.

Furthermore, the effects of plant proteins on muscle protein synthesis (MPS) were scarcely investigated in the context of appetite, a significant risk factor for malnutrition and subsequent loss of muscle tissue [27]. It has not been yet established whether plant proteins trigger similar appetite-related responses in underweight, normal weight and overweight individuals while they age. Addressing this research gap is crucial to assess whether nutritional strategies can maximise the nutritional status of an ageing adult and whether increasing protein consumption chronically reduces energy intake, thereby increasing the risk of malnutrition. More generally, insight of this kind may help consumers to make healthy food choices and will inform the development of nutritionally balanced products that promote healthy ageing. 

The purpose of this review is threefold: (1) to summarise evidence regarding the optimal quantity and daily distribution of protein intake in ageing adults; (2) to present current knowledge about sustainable protein intake in the context of appetite control; and (3) to identify the areas for future research and challenges in introducing novel food solutions to consumers.

## 2. Optimal Protein Intake

### 2.1. Daily Quantity

The current international Recommended Dietary Allowance (RDA) for protein is 0.8 g per kg of body weight (bw), regardless of age [28,29]. In the UK, the Reference Nutrient Intake (RNI) is 0.75 g/kg/bw [30]. These recommendations are derived as a minimum amount to maintain nitrogen balance and are not optimised for physical activity level (PAL). Individuals with low PAL have decreased rates of nitrogen retention and therefore in order to maintain muscle tissue have increased protein requirements in comparison to those who are active [31]. Considering that physical activity decreases with age [32], this is an important factor when protein needs are evaluated. Furthermore, the body of an ageing adult undergoes multiple physiological changes which alter protein utilisation, and thus requirements, i.e., anabolic resistance, insulin resistance, impaired digestion, inflammation, and decreased IGF-1 levels [3,10,11,12]. The adequacy of current protein recommendations has also been challenged because of potential methodological pitfalls. First, the nitrogen-balance method used in the majority of pooled studies may not be accurate, possibly due to unaccounted routes of nitrogen input and output [3,33]. A second limitation is that nitrogen-balance studies must be carried out in a controlled, clinical environment, hence the protein requirement assessment is relatively short-term [33]. Data regarding long-term evaluations of protein needs in ageing adults, with a use of novel, more accurate assessment techniques, is scarce and is identified as an academic research priority [34,35].

Acknowledging all these factors, and supported by a large body of new evidence, the International PROT-AGE Study Group [3] and European Society for Clinical Nutrition and Metabolism (ESPEN) [11] concluded that daily protein requirement of healthy individuals over 65 years is 1.0–1.2 g protein/kg/bw. A further increase is recommended for individuals with acute or chronic illnesses (1.2–1.5 g protein/kg/bw) and severe illnesses, injuries, or malnutrition (2.0 protein g/kg/bw) [3,11]. Although these new recommendations have been formulated for adults >65 years, this is only an agreed conceptual cut-off point. Since it has been estimated that 0.5–1% of muscle mass is lost annually from the age of 50 [36], an increased dietary protein intake may be required earlier in life in order to mitigate the muscle ageing process. One of the longest (14-week) interventional studies to date revealed that in adults aged 55–77, ingestion of 0.8 g protein/kg/bw was associated with decreased mid-thigh muscle area and decreased urinary nitrogen excretion (when compared to the second week of the intervention), suggesting that the current RDA might be below the actual requirements of an ageing adult [9]. The link between protein consumption at the RDA level and adverse health outcomes was also confirmed in longitudinal observational studies. As discovered by Houston and others [37], older adults (70–79 years) whose daily protein intake was 1.1 ± 0.4 g/kg/bw had lost 40% less lean body mass over the course of three years than those who consumed 0.8 ± 0.3 g/kg/bw. 

In summary, protein requirements vary on an individual basis and depend on various factors, such as age, health status and PAL. These factors are not reflected in current recommendations for the general population. Therefore, an increase in intake of dietary protein beginning around midlife merits further research

### 2.2. Daily Distribution 

Apart from the total daily intake, per-meal protein quantity and daily frequency of protein ingestion have also been shown to play an important role in preserving muscle mass and function. It is estimated that consumption of two to three meals a day, each containing ~25–30 g of high-quality protein, is optimal for the stimulation of 24-h muscle protein synthesis (MPS) in healthy adults [38,39,40,41,42]. This approximate quantity is thought to be sufficient, both for younger and healthy older adults [39]. 

Interestingly, ‘the more, the better’ approach is not necessarily optimal. Moore and colleagues [43] investigated the per-meal threshold in relation to body weight and age. In this study, protein utilisation plateaued after the ingestion of 0.24 g of whey and egg protein/kg/bw in young men (~22 years) and after 0.40 g/kg/bw in older men (~71 years) [43]. Similar findings were reported in an acute feeding study by Symons and colleagues [39] who compared the effects of 30 g of lean-beef protein/meal to 90 g/meal ingestion on MPS in young (35 ± 3 years) and older adults (68 ± 2 years) and found there was no additional benefit of increased protein consumption in either age category. The estimated per-meal threshold after consuming a plant protein-rich meal is still unknown, particularly in ageing adults [44]. 

Some studies argue that consuming a higher dose of protein on one daily occasion (pulse feeding) can stimulate a higher anabolic response than smaller doses across multiple meals [45,46,47]. Arnal and colleagues [45] reported that during the trial, women (mean age 68 ± 1 years) who consumed 79% of daily protein at noon, 7% in the morning and 14%% in the evening had improved nitrogen balance, when compared to women who consumed their protein spread over four meals (21.5 ± 0.5, 31.2 ± 0.2, 19.1 ± 0.5, 28.3 ± 0.5% of daily protein intake). However, none of these four meals contained the required bolus of 25–30 g protein per serving, which could be a potential cause why the spread pattern treatment was less effective. Reports by Bouillanne and colleagues [46,47] were in line with Arnal’s findings, suggesting that pulse feeding was more effective in improving lean mass index in older adults. However, since participants in these studies were recruited from very old (mean age 84.1 years) hospitalised patients at risk of malnutrition the results should be cautiously interpreted and cannot be generalised to younger, healthy adults. 

In most Western societies, the daily pattern of dietary protein ingestion is skewed regardless of age or sex, with the lowest amount of protein being consumed in the morning and the greatest in the evening meal [48,49,50,51]. As shown in a British cohort study that has followed the dietary intake of adults aged 36 years for 17 years, the protein content of meals has gradually shifted toward the evening [48]. Although these results refer to years 1982–1999, evidence suggests, that this trend has been sustained because it is mirrored in data collected more recently from other countries. Data from U.S. National Health and Nutrition Examination Survey (NHANES) [49] indicate that in 2006 the average protein intake (g/meal) among women aged 51–71 year age group was 11.9 ± 0.4 (breakfast), 17.9 ± 0.5 (lunch) and 30.4 ± 0.7 (dinner) with snacks constituting 7.4 ± 0.3 [49]. The intake (g/meal) in men was higher and accounted for 15.8 ± 0.5, 23.2 ± 0.8, 43.5 ± 1.0 and 10.5 ± 0.5, respectively [49]. Results from this study have also confirmed that the same pattern was observed in a ≥71 years group. However, the amount of protein consumed in each meal was lower in both sexes, in comparison to the younger age group [49]. Regarding the population of the very old community-dwelling adults, the pattern of daily protein distribution appears to peak at mid-day. The Newcastle 85+ study [52] revealed that the highest amount of protein in this British cohort was consumed at lunch time, accounting for ~35% (around 20 g) of daily protein intake, followed by dinner ~21% (12 g), and in the period between lunch and dinner ~17% (10 g). The remaining protein were consumed at two consecutive morning occasions ~22% (13 g, combined), and late evening meal.

The commonly observed uneven pattern of protein ingestion in older adults suggests a potential risk of insufficient stimulation of MPS, even when RDA on a daily basis is being met. Meaning, the stimulation with a meal containing ~25–30 g of protein occurs only once a day, during the main meal (lunch or dinner). As suggested by Bollwein and others [13], the protein distribution at older age is of higher importance than the total daily amount per se. In this study, the recommendation of 0.8 g/kg/bw was exceeded by all participants (>75 years), even those from the lowest quartile of protein intake. No differences were observed between frailty status and daily protein intake. However, those with a more uneven distribution were more likely to be classified as frail, characterised by lower walking speed and higher exhaustion [13]. Ingestion of a high-protein meal before sleep has been shown to increase overnight MPS [53], therefore this dietary habit should be sustained. In addition, to stimulate 24-h MPS, enriching the content of remaining meals with high-quality protein should be strongly encouraged, to ensure a sufficient dose of protein in each meal.

Although discussed recommendations regarding optimal protein quantity and distribution seem to be well supported, it has to be noted that most studies have analysed outcomes related solely to animal proteins and muscle health, omitting the accompanying effects of protein ingestion on appetite. Placing these findings in this context would provide valuable insight and guidance for adults who also need to limit or increase their daily energy intake in order to optimise nutritional status.

## 3. Dietary Sources of Protein

### 3.1. Protein Consumption in the UK

**Figure 1:**
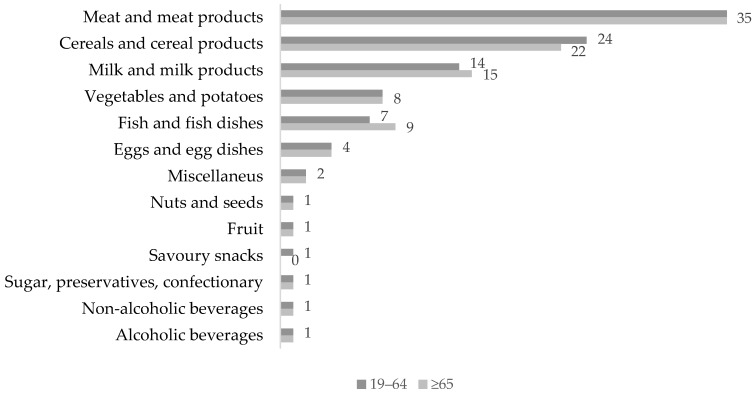
Percentage contribution (%) of food groups to average daily protein intake in the UK in 2013/2014: adults aged 19–64 and ≥65 years. Source: National Diet and Nutrition Survey Rolling Programme Years 2013–2014 [54].

Dietary proteins are found in animal-based foods, plant-based foods, and alternative sources such as algae, bacteria, and fungi (mycoproteins). Globally, plant-based foods are the leading source of protein, comprising 57% of daily protein intake, followed by meat (18%), dairy (10%), fish and shellfish (6%), and other animal products (9%) [16]. In contrast, the main source of protein in the British diet is animal-based, contributing to nearly two thirds of total daily protein intake [54]. The National Data and Nutrition Survey divides adults into two age groups (19–64 and >65 years old), which does not allow the comparison of protein intakes between more narrow age ranges (e.g., 40–65 vs. >65 years old). However, being the only nationally representative data regarding protein sources in the British diet, it was decided that it is worth the inclusion. In 2013/2014 the percentage distribution of protein intake from animal-based products in adults aged 19–64 was as follows: ‘meat and meat products’ (35%), ‘dairy’ (14%), ‘fish’ (7%) and ‘eggs’ (4%) (see Figure 1). The distribution was very similar in adults ≥65 years, although the contribution from ‘dairy’ and ‘fish’ was slightly higher than in the younger group (15%, and 9%, respectively). In the category ‘meat and meat products’ and in adults aged 19–65, the most popular foods were ‘chicken and poultry’ (13%), followed by processed meat (7%), ‘beef and veal’ (6%), ‘bacon and ham’ (4%), ‘pork’ (3%) and ‘lamb’ (2%). Plant proteins were derived mostly from ‘cereals and cereal products’ (24%)—predominantly from the ‘rice, pasta and bread’ food group (18%)—followed by ‘vegetables and potatoes’ (8%), ‘fruit’ (1%), and ‘nuts and seeds’ (1%). Again, this distribution did not differ greatly between the age groups, apart from cereals being a slightly lower contributor to the daily protein intake in adults ≥65 years (22%). The remaining 6% of protein source is difficult to classify, and comprised items such as savoury snacks, confectionary, beverages, and miscellaneous foods [54].

Alternative protein sources are not commonly consumed in Western countries. The exception is mycoproteins—primarily in the form of vegetarian meat substitutes—which have increased in popularity over the last three decades, and with a trend that is expected to continue in the future [17]. Cultured meat (‘lab-grown’) as an alternative source of protein is currently understudied. However, the future profitability and consumer acceptance of this product remains unclear [55]. 

### 3.2. Protein Quality

There is debate about the optimal source of protein and numerous quality assessment measures have been proposed [56]. The most commonly applied method to assess protein quality involves the calculation of a Protein Digestibility Corrected Amino Acid (PDCAA) Score, or Digestibility Indispensable Amino Acid (DIAA) Score [57,58]. In general, animal-based foods are recognised as a superior source of protein because they have a complete composition of essential amino acids, with high digestibility (>90%) and bioavailability [59]. Animal proteins have higher PDCAA scores than plants, suggesting greater efficiency in muscle anabolic processes [58]. For example, proteins found in milk, whey, egg, casein and beef have the highest score (1.0), while scores for plant-based proteins are as follows: soy (0.91), pea (0.67), oat (0.57) and whole wheat (0.45) [58]. However, proteins do not occur in foods in isolation and the entire food matrix should to be considered when health benefits are evaluated [56]. Apart from protein, animal-based foods provide heme-iron, cholecalciferol, docosahexaenoic acid (DHA), vitamin B12, creatine, taurine, carnosine and conjugated linoleic acid (CLA); all compounds not present in plant-based foods [60]. Thus, moderate consumption of high-quality unprocessed animal-based foods should not be discouraged entirely. On the other hand, foods of animal origin contain saturated fatty acids (SFA). Although unfavourable effects of SFA on health should not be generalised to all animal-originated products (e.g., dairy or fish oil), there is a large body of evidence that processed meat (also high in SFA) is associated with increased risk of cardiovascular disease, dyslipidaemia and some forms of cancers [61,62,63] and is classified as group 1 carcinogen [64].

Plant proteins are often described as incomplete, due to the insufficient amounts of all nine essential amino acids [59]. Although protein content and amino acid composition vary between plant species, in general, protein found in legumes are limited in methionine and cysteine; cereals (lysine, tryptophan); vegetables, nuts and seeds (methionine, cysteine, lysine, threonine); seaweed (histidine, lysine) [65]. In addition, the digestibility and bioavailability of plant proteins is lower than those from animal sources, due to the high content of dietary fibre and plant bio-compounds (also called phytochemicals), e.g., trypsin inhibitors, phytates, saponins or tannins [66]. Interestingly, regarded in the past as anti-nutritional compounds, phytochemicals are being now increasingly associated with beneficial effects, e.g., regulating blood glucose level, improving lipid profile and reducing the risk of certain cancers [67]. Moreover, the amino acid which has been shown to play an important role in MPS is leucine [68]. Leucine supplementation can increase the rate of MPS in young adults [69] and can reduce the loss of lean mass in middle-aged adults (52 ± 1 years) during periods of bed rest [70]. According to PROT-AGE recommendations, 2.5–2.8 g of leucine per meal is sufficient to reach anabolic threshold and optimise MPS [3]. In general, plants contain a lower content of leucine (<8% of total protein) in comparison to animal-originated foods (approx. 8–14%), with maize being an exception (12%) [58]. However, some plants are still a relatively good source, if consumed in larger volumes and these include: dried seaweed (4.95 g/100 g), dry-roasted soy beans (3.22/100 g), roasted pumpkin seeds (2.39 g/100 g), dry-roasted peanuts (1.53 g/100 g), and cooked lentils (1.29 g/1 cup) [71].

As suggested by other authors, solutions to maximise essential amino-acids content of plant foods include: amino-acid complementation (consuming cereals and pulses in one meal), consuming higher amounts of plant-based products on a more frequent basis or enhancing the nutritional quality of crops through genetic engineering [44,58,72]. More studies are needed to evaluate the effectiveness of plant proteins in the prevention of muscle mass and strength loss. Sources other than soy and foods or diets that are complementary in terms of amino-acid composition (e.g., composed of more than one plant) have been poorly studied.

### 3.3. Plant Proteins as a Sustainable Alternative

It is estimated that by 2030 the world’s population will reach 8.5 billion, with 1.4 billion being over 60 years old [73]. Both projected changes pose serious challenges to a food-supply system that will need to meet the nutritional needs of both an ageing and expanding population. Protein is the macronutrient most extensively discussed in the context of feeding the world for two reasons. Firstly, it plays a critical role in preventing protein-energy malnutrition (PEM) and promotes healthy muscle ageing. Secondly, global protein demand generates environmental implications associated with supplying animal-based foods; continuously the most preferable among consumers’ source of dietary protein [74]. 

Exploring alternative protein sources and transitioning towards more sustainable, plant-based diets, has been a recent research priority [16,17,24]. According to the Food and Agriculture Organisation (FAO) definition, sustainable diets have “low environmental impacts which contribute to food and nutrition security and to healthy life for present and future generations. Sustainable diets are protective and respectful of biodiversity and ecosystems, culturally acceptable, accessible, economically fair and affordable; nutritionally adequate, safe and healthy; while optimizing natural and human resources” [24]. It has been well documented that plant-based diets can lower the risk of diabetes, cardiovascular diseases, hypertension, obesity, metabolic syndrome, and mortality, as well as prevent specific types of cancer [75,76,77]. However, a rapid transformation to a vegetarian diet is unlikely to be feasible on the global scale, and it is still debatable whether it is optimal for human health, e.g., due to the risk of vitamin B12 deficiency and elevating homocysteine levels [78]. Therefore, new guidelines are being developed, promoting a mixed, yet more sustainable dietary pattern, with increased intake of plant foods and reduced intake of meat [79]. At a population level, these dietary changes, as proposed in the British ‘Eatwell Guide’, are expected to increase life expectancy, decrease disability-adjusted life years (DALYs), and decrease the incidence of diabetes, cardiovascular diseases and colorectal cancer [80]. Currently, food consumption in the UK deviates markedly from these dietary recommendations. Scarborough and colleagues [81] have modelled the changes that would be required in order to meet the recommendations outlined in the ‘Eatwell Guide’. They found that the consumption of beans, pulses and other legumes would need to substantially increase (by 85%), while the consumption of red meat and processed meat would have to fall by 75%. The ‘Eatwell Guide’ considers sustainability and endorses the partial replacement of animal proteins with plants-based proteins, such as peas, beans, legumes. However, the recommended intake of these alternative sources of proteins remains to be determined. 

## 4. Plant Proteins and Appetite Control

As concluded earlier, there is strong evidence that for optimal MPS and to prevent muscle loss an ageing adult would benefit from an increase (>1.2 g/kg/bw) in protein intake [3,11]. Ideally, proteins should be consumed three times a day with a dose of around 25–30 g of high-quality, yet sustainable protein in each meal [39,40,41,42]. However, the increased consumption of plant proteins found in whole foods (e.g., legumes, cereals, vegetables) stipulates more than one change in diet composition, i.e., apart from the increased percentage of energy yielded from proteins, dietary fibre—an integral element of all plant diets—can also be elevated considerably [82]. A diet high in both protein and fibre was demonstrated to support successful weight-loss [83]. Therefore, the incorporation of increased amounts of high-protein and high-fibre foods provides a promising strategy for overweight and obese individuals, since high-protein diets are linked to improved satiety and appetite control [84]. On the other hand, this also raises concerns about whether satiety will be enhanced by the two components, resulting in reduced appetite in individuals at risk of malnutrition. It is still unclear whether plant proteins affect appetite in the same way as animal proteins, and whether they compromise subsequent energy intake. However, it is worth emphasising that self-reported appetite is not necessarily a predictor of energy intake [85]. This section will briefly explain appetite-related mechanisms and will discuss the existing evidence regarding the potential effect of a diet high in plant proteins on appetite, across body mass index (BMI) categories. 

### 4.1. Hunger, Satiety and Appetite Mechanisms

It is important to stress that hunger and appetite are nonsynonymous terms. Hunger is defined as a physical ‘need to eat’ (usually caused by a long inter-meal interval), while appetite is a ‘desire to eat’ [86]. By contrast, satiation is a state of fullness, after hunger is suppressed [86]. Hunger and satiation are crucial elements of appetite assessment, which are usually scored using a visual-analogue scale [87]. Although a great deal of research has studied the phenomenon of appetite, underlying mechanisms remain unclear. In simple terms, hunger, satiation and appetite can be directly or indirectly stimulated by hormonal responses from: (i) pancreas, e.g., secretion of insulin, glucagon, pancreatic polypeptide (PP) and amylin; (ii) adipose tissue, e.g., leptin and adiponectin; (iii) gastrointestinal tract, e.g., ghrelin, glucagon-like peptide 1 and 2 (GLP-1, GLP-2), cholecystokinin (CKK), gastric inhibitory polypeptide (GIP), polypeptide YY (PYY), oxyntomodulin and serotonin; and iv) hypothalamus, e.g., dopamine, neuropeptide Y, growth hormone releasing peptide (GHRP) [88,89]. Some hormones or peptides promote appetite (orexigenic) and other work antagonistically, by suppressing it (anorexigenic) [88,89]. Apart from physiological factors, the sensorial exposure to food (e.g., sight, smell, taste) has been shown to increase appetite [90]. As such, people may report the desire to eat in the absence of hunger. A novel finding is that the individual’s protein status can affect the response to food cues. Griffoen-Roose and colleagues [91] discovered, that protein deprivation modulated reward responses in the brain and promoted a selective preference for savoury foods. 

Appetite control mechanisms have been suggested to be dependent on the individual’s body mass status and age [92,93]. The most consistent finding is that hunger and appetite tend to be reduced in older individuals (anorexia of ageing) [94,95]. Yet, it remains to be determined how the foods high in plant proteins influence physiological, sensorial and psychological responses, and whether they change with body weight status and age. Because of the sparsity of evidence, studies with younger participants have been included in the following section of this review.

### 4.2. Effects of Plant-Based Proteins on Appetite Control in Overweight and Obese Individuals

High protein diets have been shown to be an effective weight-loss strategy for overweight and obese individuals by reducing hunger [96]. They also produce greater satiety in comparison to carbohydrates and fats [97] and increase energy expenditure and diet-induced thermogenesis [97,98]. Most studies, confirmed the effect of high-protein diet on postprandial appetite suppression and subsequent reduction in energy intake [84,97]. To date most trials in overweight and obese adults have focused on the effects of animal proteins (e.g., meat, whey, casein) and it is unclear if the consumption of plant proteins has a similar effect on appetite. In addition, previous studies involving high-protein diets tended to use foods that are low in carbohydrate [99]. The shift towards plant-based proteins in their natural (not-isolated) form eliminates the potential ketogenic effect of a high-protein diet because these alternatives (e.g., pulses, cereals and vegetables) also tend to be rich in carbohydrate. 

When soy proteins were studied, the effects on appetite and weight loss-related outcomes appeared to be similar to those stimulated by ingestion of animal proteins. Neacsu and colleagues [100] investigated appetite responses to high-protein weight-loss diets among obese men (mean BMI 34.8 kg/m^2^), aged 34–71 years old. The study demonstrated that weight loss was observed in both (meat and soy) diets, and the magnitude of weight loss did not differ significantly between the intervention groups. No significant differences in hunger, fullness or desire to eat were observed between the participants following diets with different protein sources. Although plasma concentration of ghrelin and PYY differed slightly between the diets, the net area under the curve (AUC) revealed that the response patterns were similar [100]. However, observations in this study were limited to soy proteins. 

Vegetarian protein sources, other than soy, were studied by Scully and colleagues [101] who compared effects of buckwheat and fava bean protein on appetite in participants aged 23–63 years old (BMI 19.3–38.9 kg/m^2^). The results revealed no significant differences in terms of motivation to eat or appetite in comparison to the baseline, and between the two diets studied. This suggesting that a shift to plant proteins is unlikely to compromise appetite of normal, overweight, and obese individuals [101]. However, in this study the age and BMI of participants differed considerably, and it would be interesting to analyse these effects in a larger and more homogeneous sample. 

In contrast, some studies have reported the differences between animal and plant proteins in terms of energy expenditure (EE) and thermogenesis. For example, Mikkelsen and others [102] found that pork protein generated a 2% higher 24 h EE than the soy protein diet, in overweight men (mean age 26 ± 3.2 years; BMI 28.9 ± 1.7 kg/m^2^). This was suggested to be due to the higher protein-nitrogen ratio of animal proteins. 

### 4.3. Effects on Plant-Based Proteins on Appetite Control in Normal Weight Individuals

Plant proteins (similarly to animal proteins) have been shown to induce fullness more effectively in normal weight subjects than in obese individuals, even when plant sources other than soy were tested. This can be explained by possible impairments in appetite control mechanisms observed among people with higher BMI [103,104]. An interesting study by Nilsson and others [105] investigated the effects of an evening meal composed of brown beans on appetite-regulating hormones in young adults (23.8 ± 0.7 years; BMI 22.5 ± 0.6 kg/m^2^). They found a significant increase in PYY (by 51%) and decrease in ghrelin and hunger feeling (by 15 and 14%, respectively), when compared to the reference meal (white wheat bread). Although the observed responses were believed to be induced by the colonic fermentation caused by the starch found in brown beans, a protein-induced satiety response cannot be entirely ruled out. 

Several studies did not find significant differences between animal and plant proteins in terms of appetite control in normal weight adults. Lang and colleagues [106] compared satiating effects of egg albumin, casein, gelatin, soy, pea and wheat gluten protein among young, healthy men. No significant differences were found between protein source and satiety, subsequent energy intake and insulin secretion. The authors suggested that carbohydrate and fat content of experimental meals may have affected the observed responses. A year later, the same research group reported different effects of casein, gelatin and soy protein ingestion on glucose, insulin and glucagon kinetics [107]. No effects were reported in terms of protein source and 24 h energy intake and only a weak effect of protein type on satiety was shown. Similar findings were reported by Douglas and colleagues [108], who compared the effects of two high-protein meals (beef vs. soy) on appetite, satiety and food intake in young adults (mean age 21 ± 1 years; BMI 23.4 ± 0.6 kg/m^2^). To account for potential confounders, two types of meals were compared: macronutrient and fibre-matched (24 g of either beef or soy protein), and size-matched (beef: 24 g protein/1 g fibre; soy: 14 g protein/5 g fibre). Under both conditions studied, fullness and postprandial PYY and GLP-1 plasma concentration increased as anticipated. However, no differences between meat and soy protein ingestion were observed and, importantly, no differences were observed in subsequent energy intake and the time when the next meal was requested [108]. In contrast, a recent study in young, normal weight men observed, that high protein plant meal (beans and peas) resulted in lower appetite, hunger and food consumption and higher fullness and appetite, when compared to meat-based meal (veal and pork) [109]. However, this finding is restricted to young adults only. In summary, results indicate that high-quality proteins, regardless of the source, have similar effect on appetite in normal weight adults and could be therefore be used interchangeably.

### 4.4. Effects of Plant-Based Proteins on Appetite Control in Underweight Individuals

To our knowledge, no study has thoroughly investigated the effects of various plant proteins on appetite in underweight, ageing adults. Moreover, it remains unclear whether protein-induced satiety decreases with age. Since soy proteins have been shown to suppress appetite in a similar way to animal proteins in normal weight and overweight subjects, this vegetarian alternative may not be optimal for adults who are older or at risk of malnutrition. Protein found in other pulses, cereals or nuts might be the answer, yet this research field is still lacking sufficient evidence. Food solutions for this population group appear to require separate investigation; perhaps a form of food, rather than the protein source, is of greater importance, e.g., few studies have shown that proteins in a liquid form, suppress appetite less than solid foods [110,111]. Hence, products targeting this population group could be in the form of high-protein soups, puddings or smoothies. Undoubtedly, high-quality, sustainable protein sources, effective in MPS stimulation, which at the same time do not compromise appetite, are highly sought after and should be considered a priority for future research.

In summary, data regarding the effects of plant proteins on appetite in adults with different body weight status is limited. The message from the existing evidence points towards the hypothesis that plant proteins trigger similar responses to animal proteins, particularly when soy proteins were studied. The appetite suppression effect is observed mainly in normal weight and overweight individuals. The appetite suppression response in underweight subjects appears to be somewhat reduced when animal proteins are ingested [112,113], which allows a speculation that increased intake of plant proteins should not mitigate energy intake at individuals at risk. This, however, needs to be verified in future studies. Other factors that affect appetite are still currently being studied, e.g., FTO gene polymorphism. One study by Huang and colleagues [114] revealed that people with the specific allele of this gene may respond differently to high-protein diets, presenting lower food cravings and appetite than people without this polymorphism. It is possible, that more research in this emerging field will advance the understanding of protein-appetite associations and perhaps explain the inconsistencies in results reported to date. 

## 5. Areas for Future Research

### 5.1. Consumer-Focused

More research is needed to explore how to build consumer awareness about the importance of sufficient protein intake for healthy ageing. Currently, high-protein foods are mainly targeted to athletes and those who aim to lose weight. The market offer of real food solutions is still modest, with the majority of high-protein products being enriched with protein derived from dairy (e.g., whey, casein). The critical question is whether the increased intake of protein for muscle health will be in the future promoted among the general population of ageing adults. For example, will this message be supported by policy makers and national guidelines, in a similar manner to the salt and sugar reduction recommendations. Moreover, the current EU labelling regulations prevent indicating the potential health benefits of the high-protein product content (e.g., health claims). Hence, today’s consumers may lack the essential knowledge of the potential health benefits associated with high-protein product consumption.

Furthermore, it would be interesting to learn more about consumer’s attitudes towards increasing the consumption of sustainably sourced proteins: (i) whether they are ready to make more environmentally friendly choices by replacing animal proteins with those from plants [115] and (ii) do they have the knowledge and cooking skills that allow for the incorporation of various plant proteins into their everyday diet? Perhaps, at the introductory phase, ready meals and snacks high in plant proteins would be a preferred consumer choice. While answering these questions it would be worthwhile to investigate potential acceptance and effectiveness of two routes: new product development (NPD) and product reformulation. The latter, could be achieved through a ‘health by stealth’ strategy which has been shown to be successful elsewhere, e.g., gradual reduction of salt in products aimed at children [116]. In the protein scenario, animal-based ingredients in commonly consumed products could be gradually and partially replaced with plant alternatives, giving the consumer time to adjust to new flavours, smells, and textures.

Lastly, more evidence regarding age-, BMI- and sex-related differences in appetite responses to plant protein meals is needed. Most studies to date were conducted in young men or young mixed-sex samples [100,101,102,105,106,108] with no comparisons between sexes being drawn. It was previously reported, that hunger, satiety and appetite responses are different in women and men [117]. To our knowledge, no studies have yet investigated the effects of a high-protein plant diet on appetite, accounting for sex differences in adults from different age groups and with different nutritional status. 

### 5.2. Industry-Focused

One of the key identified challenges in new product development is the palatability of foods high in plant proteins. The pleasantness of the diet is an interesting area of appetite and satiety research. It has been shown, that apart from the self-perceived hunger/fullness and postprandial hormones secretion, a central nervous system response to a high protein meal ingestion is of an equal significance [118]. Although the results are inconclusive, in general, meals high in animal proteins are scored higher on palatability scales than high-protein vegetarian alternatives [109]. However, it has been suggested that the regular exposure to meat alternatives can positively influence a product’s liking over time [119]. It would therefore be important to explore and evaluate potential methods to increase the palatability of plant-based foods.

Furthermore, the food industry would need to address the product design challenge related to the incorporation of the required amount of 25–30 g of high-quality plant proteins into one meal. To maximise the benefits of the natural food matrix, this would need to be achieved while preserving a sensible volume size, and preferably without the use of isolates and concentrates. Lastly, it would be worthwhile to assess which sustainable (yet nutritious) protein sources would be most feasible to grow and produce in the UK. Wheat and barley are one of the most commonly grown crops in Britain [120]. Schoeder and colleagues [121] compared the effect of barley, rice and wheat on appetite and found that while no significant differences were observed in terms of subsequent energy intake, a high-fiber barley snack significantly reduced hunger sensation in comparison to rice and wheat. Soy proteins were the most extensively studied type of vegetarian protein and other types of plant proteins have not been yet thoroughly investigated.

## 6. Conclusions

The currently recommended protein intake for ageing adults may not be sufficient for muscle mass and strength maintenance. To minimise the adverse health and environmental effects of excess animal protein consumption, incorporation of sustainably sourced plant proteins may be a promising strategy. Unfortunately, healthy and environmentally friendly food solutions are still in the conceptual phase and require more supportive research-based evidence. Although the evidence regarding the effects of plant proteins on appetite is scarce, available data points towards the positive effects of replacing animal proteins with plant-originated protein in normal weight as well as overweight/obese individuals. More studies are needed to understand the effect of these protein sources on satiety, in underweight adults.
